# Statistical analysis, source apportionment, and toxicity of particulate- and gaseous-phase PAHs in the urban atmosphere

**DOI:** 10.3389/fpubh.2022.1070663

**Published:** 2023-01-10

**Authors:** Bhupendra Pratap Singh, Torki A. Zughaibi, Saif A. Alharthy, Ahmed I. Al-Asmari, Shakilur Rahman

**Affiliations:** ^1^Department of Environmental Studies, Deshbadhu College, University of Delhi, New Delhi, India; ^2^Delhi School of Climate Change and Sustainability, Institute of Eminence, University of Delhi, New Delhi, India; ^3^Department of Medical Laboratory Sciences, Faculty of Applied Medical Sciences, King Abdulaziz University, Jeddah, Saudi Arabia; ^4^Toxicology and Forensic Science Unit, King Fahd Medical Research Center, King Abdulaziz University, Jeddah, Saudi Arabia; ^5^Laboratory Department, Ministry of Health, King Aziz Hospital, Jeddah, Saudi Arabia; ^6^Department of Medical Elementology and Toxicology, School of Chemical and Life Sciences, New Delhi, India

**Keywords:** PAHs, PCA, correlation, seasonal variation, carcinogenic health risk

## Abstract

**Introduction:**

The concentrations of particulate and gaseous Polycyclic Hydrocarbons Carbon (PAHs) were determined in the urban atmosphere of Delhi in different seasons (winter, summer, and monsoon).

**Methodology:**

The samples were collected using instrument air metric (particulate phase) and charcoal tube (gaseous phase) and analyzed through Gas chromatography. The principal component and correlation were used to identify the sources of particulate and gaseous PAHs during different seasons.

**Results and discussion:**

The mean concentration of the sum of total PAHs (TPAHs) for particulate and gaseous phases at all the sites were found to be higher in the winter season (165.14 ± 50.44 ng/m^3^ and 65.73 ± 16.84 ng/m^3^) than in the summer season (134.08 ± 35.0 ng/m^3^ and 43.43 ± 9.59 ng/m^3^), whereas in the monsoon season the concentration was least (68.15 ± 18.25 ng/m^3^ and 37.63 1 13.62 ng/m^3^). The principal component analysis (PCA) results revealed that seasonal variations of PAHs accounted for over 86.9%, 84.5%, and 94.5% for the summer, monsoon, and winter seasons, respectively. The strong and positive correlation coefficients were observed between B(ghi)P and DahA (0.922), B(a)P and IcdP (0.857), and B(a)P and DahA (0.821), which indicated the common source emissions of PAHs. In addition to this, the correlation between Nap and Flu, Flu and Flt, B(a)P, and IcdP showed moderate to high correlation ranging from 0.68 to 0.75 for the particulate phase PAHs. The carcinogenic health risk values for gaseous and particulate phase PAHs at all sites were calculated to be 4.53 × 10^−6^, 2.36 × 10-5 for children, and 1.22 × 10^−5^, 6.35 × 10^−5^ for adults, respectively. The carcinogenic health risk for current results was found to be relatively higher than the prescribed standard of the Central Pollution Control Board, India (1.0 × 10^−6^).

## Introduction

In the last few decades, urban air pollution has become a serious environmental problem, especially in developing countries, including India (1–4). Widespread industrialization, rapid urban planning, and a large increase in the number of vehicles with a high population density have been responsible for a deterioration in the ambient air quality (5–8). Among air pollutants, polycyclic aromatic hydrocarbons (PAHs) are among the most important due to their impact on both health and climate (9–11).

Polycyclic aromatic hydrocarbons are a group or class of hydrocarbons with multiple aromatic rings fused in various configurations that appear to have a universal presence in the environment and are the first atmospheric pollutants whose carcinogenic and mutagenic nature has been assessed (12, 13). Several studies reported that incomplete combustion of fossil fuels contributed to approximately 60% of the global emission of PAHs (14–16). The emission of PAHs to the atmosphere comes from both natural and anthropogenic sources. The emission of PAHs from natural sources is combustion from forest fires and volcanic eruptions (17, 18), whereas anthropogenic sources are due to incomplete combustion of fossil fuels (coal, wood oil, diesel, and petrol) at high temperatures (12, 19–22). Several studies reported that high concentrations of PAH were also found in petroleum products, coal tar, crude oil, creosote, and roofing tar (23–25). The partitioning of PAHs into a particular gaseous phase is determined by the molecular weight of the compounds as well as the meteorological parameters (26).

The principal sources of PAHs are the incomplete combustion of fuels and other organic substances, which contribute in the range of 70–90% (27). Many studies have pointed out that the levels, human exposure, and composition may vary by geographical area (12, 28). PAHs are the products of incomplete combustion and domestic activities, which contribute to ~ 60% of global emissions of PAHs into the environment (29). Naturally, PAHs can be eliminated by hydrolysis, biodegradation, and photolysis so that the concentration of PAHs in the environment is always maintained in dynamic equilibrium (27).

Currently, the widespread distribution of PAHs in the atmosphere is of great concern to scientists, which has led to their critical study for proper monitoring of concentration and release into the environment (30). Bioaccumulation of PAHs is highly influenced by the particle phases in the atmosphere and their partitioning between the gaseous phases (13, 31, 32), and the most dominant forms of PAHs that exist in the environment are the particulate and gaseous phases (33). The most common PAHs associated with particulates were pyrene, phenanthrene, acenaphthylene, and fluoranthene, which were associated with diesel and gasoline exhaust particles. PAHs with a low ring structure exist only in the gaseous form (33–35), while PAHs with a high ring structure are mainly associated with the particulate form, which adsorbs on the surface of particles in large amounts (36).

Several studies reported that PAHs are considered to be carcinogenic and mutagenic agents (32, 37), even in India with a high concentration of PAHs with potential exposure risks (38–40). Moreover, long-term exposure to PAHs may cause damage to our human cell lines, cardiopulmonary mortality, and pulmonary tissue damage (14, 29, 41). In addition, a variation in the health risks caused by PAHs has been seen among different age groups and different genders. Several studies showed that the risks of cancer caused by PAHs are ~ 4.83 times higher in adults than those in children through the inhalation pathway due to their longer exposure time and the larger body weight (42). In addition, several literature studies associated PAHs with various diseases, including cardiovascular diseases, bone marrow diseases, immune system suppression, liver diseases, reproductive diseases, and cancer (18, 43, 44).

Based on the aforementioned assessment of PAH levels, especially in the gaseous phase in the urban ambient atmosphere, fuel consumption from transport (driven by petrol and diesel) is attributed as a predominant source of PAHs (10). Few studies in the literature have assessed particulate-phase PAHs, resulting from the gaseous phase, and their correlation is limited in the scientific literature. Therefore, this current study aimed to evaluate the different levels of particulate- and gaseous-phase PAHs in the urban city of Delhi, with the following objectives: (a) to compare particulate- and gaseous-phase PAHs in different seasons, (b) to determine the source apportionment of PAHs using different statistical analyses, and (c) to estimate the health risk assessment of particulate- and gaseous-phase PAHs with exposure to different age groups.

## Methods and materials

### Sampling area

In this study, five topographical sites in Delhi were identified for the study of PAH concentration in ambient air. These sites included JNU, Mukherjee Nagar, Rohini, Anand Vihar, and CP. The basis of this selection included land use and its pattern of coverage, the number of automobiles, the presence of electricity, and safety. The details of sampling coordination and meteorological parameters are presented in Supplementary Tables 1, 2. In total, 96 samples were collected from each monitoring station for particulate and gaseous emissions.

### Monitoring of particulate-phase and gaseous-phase PAHs

For the particulate-phase PAHs, an air sampler (Airmetrics Minivol) was used for sampling. This device was operated with a reusable battery, a 24-h backup, and a low consumption rate. It maintains a 5 L/min flow rate to ensure steady performance throughout the sampling period of the impactors, which are fitted at 1.5 m above the second floor of household apartments at every chosen location. The air sampler collected PM_2.5_ on a 47-mm polytetrafluoroethylene (PTFE) filter sheet (45).

The gaseous phase of PAHs was collected on an absorbent tube (ORBO^TM^) with a polyurethane foam (PUF) plug and glass cassettes with XAD-2 resin. Most scholars claim that this resin shows greater efficiency in the separation of naphthalene (46). The fluidity rate of the samples was taken using a rotameter (accuracy ±1%). Then, the samples were covered with a silver foil, stored in a very clean screw-capped vial using a Teflon cap liner, and then placed in refrigerated containers (4°C temperature) for further transport.

Ambient air samples through both XAD-2 and the filter were kept at room temperature to warm them. The resin from the XAD-2 tubes was placed in 4-ml screw-top vials. The front and back sections of the XAD-2 resin were placed in different vials and labeled front and back with a marker. The PTFE filter was first used to cut the samples into small pieces, and they were also placed in separate 4-ml screw-top vials. In each vial, 2 ml of methyl chloride was added and shaken for 2 min. Laboratory and field blanks were also extracted in the same way. From each vial containing XAD resin or filter, 1 ml of the extract was transferred to an autosampler vial for further analysis by gas chromatography/mass spectrometry (GC/MS). The analysis was carried out on a Bruker 450GC (gas chromatograph) equipped with a DB-5 capillary column (30 m × 0.25 mm × 0.25 μm film thickness). According to the procedures listed by the National Institute for Occupational Safety Health (NIOSH) Method 5515, the analysis of PAHs in air samples was performed (47). The details for the extraction and chemical analysis method are presented in Supplementary Table 1.

## Method validation

Several studies suggested the calculation and validation methods for PAH concentration, which include various parameters such as linearity, recovery, precision, limits of detection (LOD), and limits of qualification (LOQ). In this study, linearity was estimated through spiked calibration levels, ranging between 10 and 500 ng/l. To estimate the recovery accuracy, three spiked blank samples were prepared at different concentration levels of 25, 50, and 200 ng/l. LOD and LOQ were calculated according to the sample PAH concentration at a signal-to-noise ratio of 3–10. The amount of PAHs in particulate- and gaseous-phase samples was estimated by interpolating the peak areas of each PAH to the internal standard peak area in the sample (Supplementary Figures 1, 2).

## Principal component analysis

Principal component analysis (PCA) is one of the important tools that changes a set of observations of possibly linked variables into a set of values that are not linked. In this study, PCA was performed at five different monitoring stations in Delhi to determine the relationship between PAHs and to identify the causes of ambient air pollution. The PCA process was used to identify the source contribution based on the variability of the measured element in a large number of samples. PCA results indicate which factors can explain the main part of the data variance (24). PCs are the eigenvectors of a covariance matrix or a correlation matrix, and each PC extracts a maximal share of the total variance. A PC with an eigenvalue greater or equal to 1 is considered statistically significant (48). In this study, factor loading, the percentage variance, and the cumulative percentage are explained by each factor and each component for the data obtained. In addition, the following sources of PAHs have been incorporated from various literature sources that use the PCA method to increase the accuracy of emission source identification (24, 48, 49).

## Health risk calculation

In this study, B(a)P is considered as a reference to calculate the toxicity equivalent factor (TEF) of all PAHs. The toxicity equivalent concentration (TEQ) of PAH equation broadening performed for health risk assessment can be calculated as described below (33, 35, 50):


(1) $$\begin{array}{l} TEQs=\sum C_{i}\;\times \;TEF_{i} \end{array}$$

Here,

*C*_*i*_ = level of PAHs.

TEF_*i*_ is the amount of toxic equivalence of samples.

Health assessments were carried out in previously published studies (10, 33).

The incremental lifetime cancer risk (ILCR) was estimated as the risk of exposure to chemicals suspected to have carcinogenic effects based on the USEPA standard models (51–53). ILCR was calculated based on the corresponding lifetime average daily dose (LADD) of PAHs by considering two different age groups: children (age 6 years) and adults (age 52 years). LADD indicates the amount of PAH intake per kilogram of body weight per day. LADD and ILCR were estimated in Equations 2 and 3, respectively.


(2) $$\begin{array}{l} LADD\;\left(mg\;kg^{-1}day^{-1}\right)\;=\;\left(Cs\;\times \;IR\;\times \;CF\;\times \;EF\;\times \;ED\right)\;/\\ \left(BW\;\times \;AT\right)Cancer\;risk\;\left(ILCR\right) \end{array}$$

(3) $$\begin{array}{l} Cancer\;risk\;=\;LADD\;\times \;CSF\;\left(Slope\;Factor\right) \end{array}$$

where Cs is the total of converted amount of PAHs based on toxic equivalents of BaP (ng m^−3^) using the toxic equivalency factor (TEF) value. IR is the air inhalation rate (m^3^ day^−1^) (53), CF is the unit conversion factor (1 × 10^−6^ mg kg^−1^), EF is the exposure frequency (day year^−1^), and ED is the exposure duration (day years^−1^) (54). ED is the value for children (6 years) and adults (52 years). BW represents body weight (kg) (53). AT represents the carcinogen averaging time (days) (55), and CSF represents the inhalation cancer slope factor (3.85 mg kg^−1^ day^−1^).

## Statistical analyses

Statistical analyses, such as factorial analysis and correlation, were performed using Statistical Package for the Social Sciences (SPSS) version 26.0 (SPSS, Inc., Chicago, IL, USA). Factorial analysis and correlation were performed to identify the correlated variables in different seasons for both particulate- and gaseous-phase PAHs in the ambient atmosphere.

## Results and discussion

### Seasonal variation in particulate- and gaseous-phase PAHs

In this study, 14 out of the 16 PAHs were identified as having a higher molecular weight associated with the particulate phase, while low molecular weight PAHs (acenaphthylene and acenaphthene) were not detectable in particulate-phase PAHs. Similarly, 8 out of the 16 PAHs were identified as having a low molecular weight associated with the gaseous phase, while high molecular weight PAHs [Chr, B(a)A, B(k)F, B(b)F, B(a)P, IcdP, DahA, and B(ghi)P] were not detectable in gaseous-phase PAHs.

The amount of total PAHs (TPAHs; particulate and gaseous phases) in all areas was higher in the winter season (165.14 ± 50.44 and 65.73 ± 16.84 ng/m^3^) than in the summer season (134.08 ± 35.0 and 43.43 ± 9.59 ng/m^3^), whereas in the monsoon season, the concentration was lower (68.15 ± 18.25 and 37.63 ± 13.62 ng/m^3^), as similar results were obtained in Delhi (India) by Singh et al. (56). A study conducted in eastern India reported a much higher average annual PAH concentration, ranging from 797.9 ± 39.1 to 1,015.1 ± 42.7 ng/m^3^ compared to the present study (57). Gaseous-phase PAHs showed less significant variation during the different seasons due to more local sources of origin, whereas particulate-phase PAHs might be local but could translocate away from the emission site. Some barometric factors also played a significant role in controlling the concentration of PAHs in all areas; at the same time, area-specific emission sources might have influenced their concentration in the surrounding atmosphere (10, 58, 59). Several studies reported a much lower TPAH concentration compared to the present study, such as 70.4 ng/m^3^ in Italy (60), 39.5 ng/m^3^ in La Plata, Argentina (59), and 20.9–65.4 ng/m^3^ in Spain (61).

The availability of more PAHs in the sample over Anand Vihar during the winter was due to the lower amount of photochemical destruction, the restricted mixing layer, and the continuous production of the temperature inversion layer. During the hot season and monsoon, a higher amount of photochemical destruction and mixture layers in the atmosphere might result in a lower PAH concentration in the samples, and also humidity and precipitation might play an important role during the monsoon period (10).

**Figure 1:**
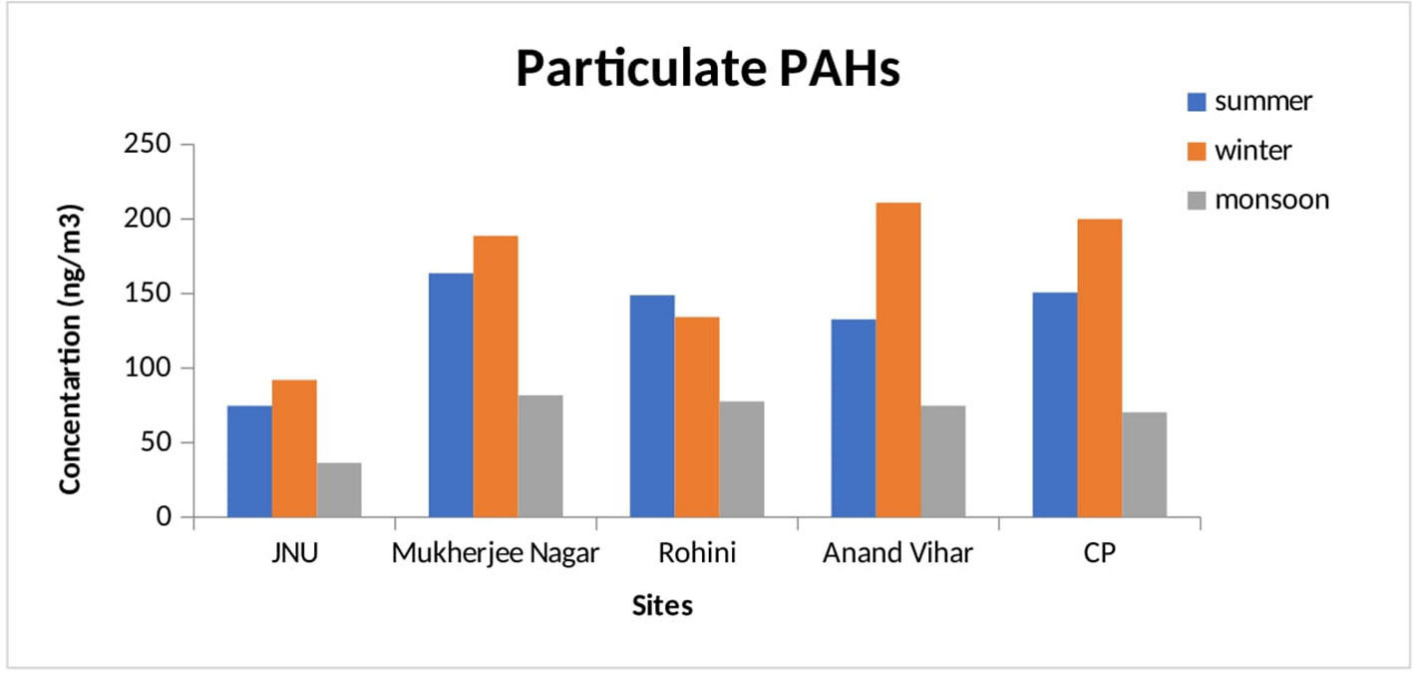
Seasonal variation of particulate PAHs at different monitoring stations.

**Figure 2:**
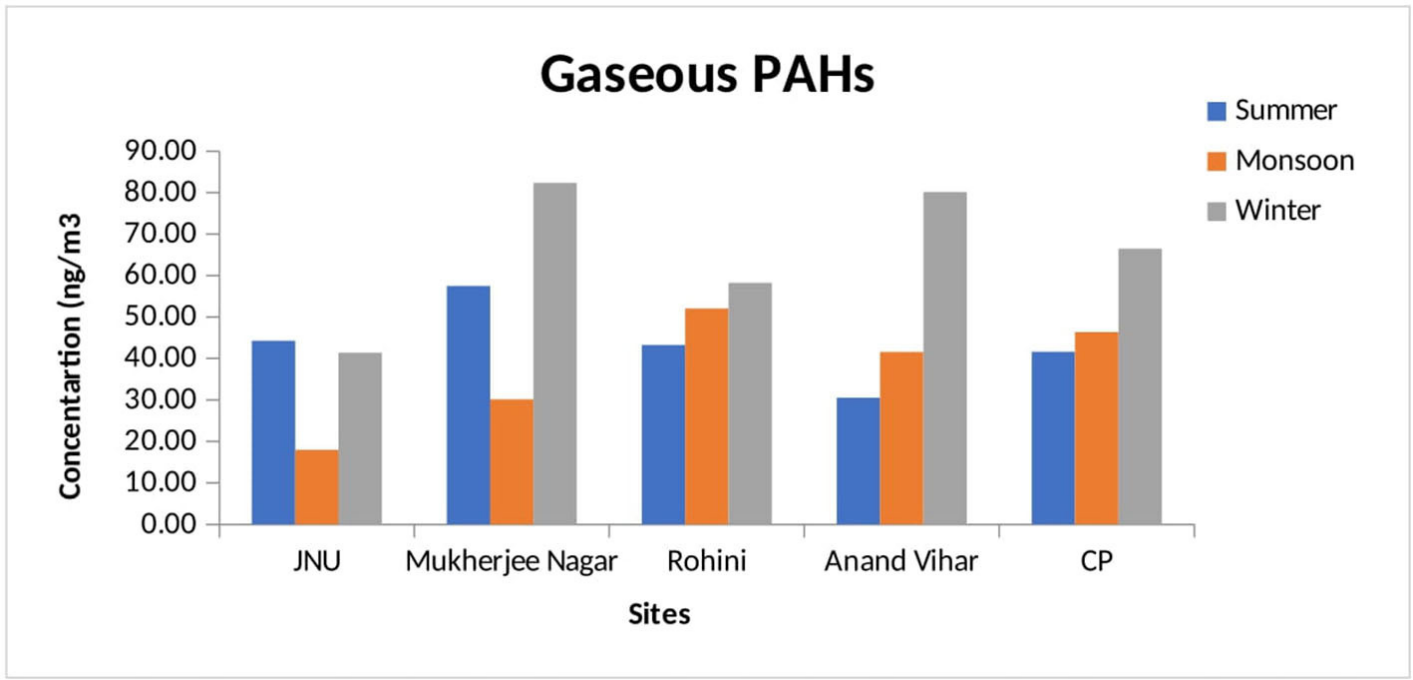
Seasonal variation of gaseous PAHs at different monitoring stations.

In the winter season, particulate-phase TPAHs were found to be higher as compared to gaseous-phase PAHs in the winter season. It ranged from 91.99 ± 6.51 (JNU) to 210.94 ± 14.30 ng/m^3^ (Anand Vihar) for particulate-phase PAHs but from 41.40 ± 1.19 (JNU) to 82.37 ± 8.0 ng/m^3^ (Mukherjee Nagar) for gaseous-phase TPAHs in the winter season. In the summer season, the amount of particulate-phase TPAHs was reported to be higher as compared to gaseous-phase PAHs. It ranged from 74.64 ± 5.03 (JNU) to 163.61 ± 9.17 ng/m^3^ (Mukherjee Nager) for particulate-phase PAHs but from 30.53 ± 1.90 (Anand Vihar) to 57.47 ± 2.51 ng/m^3^ (Mukherjee Nagar) for gaseous-phase PAHs. Further, in the monsoon season, it ranged from 36.34 ± 2.80 (JNU) to 81.70 ± 5.84 ng/m^3^ (Mukherjee Nager) for particulate-phase PAHs, whereas it ranged from 17.97 ± 2.25 (JNU) to 52.06 ± 6.51 ng/m^3^ (Rohini) for gaseous-phase PAHs. The trend of the maximum concentration of the particulate phase was in the following order: Mukherjee Nagar > Anand Vihar > CP > Rohini > JNU during the winter season. Apart from the summer season, a pattern for the highest amount of the particulate phase was in the sequence of Rohini > Mukherjee Nagar > Anand Vihar > CP > JNU and that of gaseous-phase PAHs was in the sequence of Mukherjee Nagar > CP > JNU > Rohini > Anand Vihar. The mean TPAHs level at all monitoring stations has presented for summer, monsoon, and winter seasons in Figures 1, 2.

The level of particulate-phase PAHs was observed to be relatively higher at Mukherjee Nagar and Anand Vihar sites than at the other monitoring sites. The Anand Vihar site is considered to be an interstate bus terminal, which indicates higher vehicular emission sources. Additionally, other PAH emission sources in Delhi certainly had large seasonal variations, including residential biofuel burning and open burning of biomasses (62). Excessive traffic during the winter period due to fog and haze was also responsible for increasing the atmospheric level of PAHs during winter (63). The recorded TPAH concentration was low in summer due to photochemical degradation and dispersion of PAHs in Delhi (64), whereas the highest concentration was observed in the winter season as a common phenomenon in many urban residential areas (65).

## PCA for annual PAHs

Principal component analysis for annual PAHs was calculated as a mean value for all seasons during the sampling period, as presented in Supplementary Tables 3, 4 for particulate- and gaseous-phase PAHs. Annually, for the particulate phase, six principal components (PCs) were extracted, while for the gaseous phase, three factors were extracted at different sites. In this study, a high factor loading for these PAHs was obtained in PC-1 for particulate-phase PAHs [B(a)P, B(k)f, B(g,h,i)P, and endo(cd)pyrene]. The present result indicated that PC-1 (eigenvalues 5.61) of particulate-phase PAHs represented gasoline sources. A similar result reported gasoline emission sources for B(a)P, B(k)F, B(g,h,i)P, and endo(cd)pyrene (66). Another study in east-central India reported higher PAH rings associated with diesel emission and coal combustion sources (57). Several researchers suggested that diesel emissions from vehicles had a high factor loading for fluorene, phenanthrene, anthracene, and pyrene (67), whereas Zhao et al. (68) proposed that fluorene and phenanthrene with a high factor loading of benzo (b & k) fluoranthene indicated diesel-driven vehicles. Several studies reported that diesel emissions from road traffic were associated with low and medium molecular weight (three to four rings) PAHs (69).

In this study, a high factor loading for these PAHs has been obtained in PC-4 for particulate-phase PAHs. Thus, it was witnessed that PC-4 of particulate-phase PAHs represented diesel-driven sources. Annual PCA revealed for the gaseous phase that Nap, Acy, Ace, and Flu were dominant species in the first factor, which indicated natural sources of emission for all seasons. The initial factor for most of the total variance (35.0%) was high loading with B(b)P, B(k)P, B(a)P, IcdP, DahA, and B(ghi)P for the particulate phase, whereas the gaseous phase accounted for the total variance (34.4%) with Nap, Acy, Ace, and Flu. The first factor with B(b)P and B(ghi)P confirmed that vehicle emission from traffic was one of the significant sources of the PAHs in all three seasons for the particulate phase (70). The second factor accounted for 23.17% of the total variance, where Anth and Pyr were identified. This factor accounted for natural gas sources (71). It reflected a substantial influence of low molecular weight PAHs with low rings (three to four rings PAHs). Several studies reported that high-loading Phe was associated with either unburned petroleum from vehicles or coal combustion (72, 73). A study was conducted on the gaseous phase reported that the first factor accounting for the majority of the total variance (25%) was highly loaded with BaP and DahA, while factor 2 was a high-loading factor with Phe and Flt (24).

## PCA for seasonal PAHs

Principal component analysis for particulate-phase PAHs extracted three factors for all seasons (summer, monsoon, and winter). The seasonal variations of PAHs accounted for more than 86.9%, 84.5%, and 94.5% for the summer, monsoon, and winter seasons, respectively, as a similar result in Changsha, China, was reported for 85.8% and 89.9% of the total data variance in the summer and autumn samples, respectively, for the particulate phase (74), while this study accounted for 83.6%, 75.5%, and 82.4% for gaseous phase samples in the three seasons. It was reported that similar PCA results in Japan accounted for 88.6% of the variance with a high loading of all PAHs, which indicated traffic emission (49).

In the summer season, for the particulate phase, the first factor illustrated 71.8% of the total variance, which indicated the loading of higher molecular weight PAHs, such as B(k)P B(a)P, DahA, and B(ghi)P. Transport was validated to be a significant contributor to higher PAHs. The loadings of lighter PAH (Acy, Ant, and Flu) for the particulate phase and Nap, Acy, and Flu for the gaseous phase were also higher for this factor, which accounted for natural gas sources (46). The second factor accounted for 18.4% of the total variance, with a finding of loading for Chry PAH as dominant, which may have been emitted from petrol and CNG vehicles for particulate-phase PAHs, whereas the gaseous phase accounted for 21.5% of the total variance and dominant species were Chry and B(a)P, which may have been emitted due to gasoline emission (75, 76).

During the monsoon season, the first factor demonstrated 51.7% of the total variance, which indicated the loading of higher PAH [such as DahA, BghiP, and B(a)P] for the particulate phase and PAHs such as Anth and Phen for the gaseous phase, respectively (Table 1). This study indicated that diesel and gasoline emission sources were significant sources (66). The second factor explained 17.8% and 24.0% of the total variance for particulate- and gaseous-phase PAHs, respectively, during the sampling period. The loading of higher PAHs Nap [B(a)P, and B(k)P] for the particulate phase and Nap, Acy, and Ace for the gaseous phase were the dominant species attributed to natural gas sources (24).

**Table 1:** Result of factor analysis with varimax rotation for particulate phase PAHs at different seasons.

**PAH**	**Summer**			**Monsoon**			**Winter**		
	**1**	**2**	**3**	**1**	**2**	**3**	**1**	**2**	**3**
Nap	−0.061	0.799	−0.582	0.720	−0.020	−0.589	−0.44	−0.73	0.25
Acy	0.826	0.396	−0.373	–	–	–	–	**–**	–
Ace	−0.945	0.306	0.086	–	–	–	–	**–**	–
Flu	0.864	−0.270	0.344	0.462	−0.528	–	–	**–**	–
Phe	−0.894	0.297	−0.313	−0.529	0.421	0.572	0.01	**0.94**	0.21
Ant	0.948	0.316	0.028	0.850	0.216	0.368	**0.95**	−0.14	−0.24
Flt	0.836	0.546	−0.025	0.484	−0.677	0.244	**0.94**	−0.16	−0.14
Pyr	0.708	0.590	0.349	−0.789	−0.280	−0.182	−0.45	0.86	−0.10
B(b)A	**0.971**	−0.050	0.114	−0.170	**0.810**	−0.086	−0.87	−0.06	0.46
Chry	0.192	**0.908**	0.366	−0.960	−0.021	−0.238	**0.89**	0.04	−0.37
B(b)F	−0.763	0.482	0.430	0.319	**0.807**	−0.398	0.02	−0.69	**0.70**
B(k)F	**0.996**	−0.038	0.043	−0.286	−0.122	0.825	0.31	−0.94	0.11
B(a)P	**0.981**	0.050	−0.182	**0.917**	0.287	0.132	0.07	0.19	**0.97**
IcdP	**0.960**	−0.094	−0.263	−0.934	0.190	−0.158	0.38	−0.14	**0.88**
DahA	**0.947**	−0.282	−0.142	**0.973**	0.041	0.107	0.55	0.54	0.59
BghiP	**0.972**	−0.081	0.201	**0.928**	0.112	0.121	0.66	0.46	0.57
Initial Eigenvalues	11.500	2.950	1.320	7.25	2.50	2.09	4.72	4.13	3.44
% of variance	71.88	18.47	8.25	51.7	17.83	14.90	36.29	31.74	26.48
Cumulative %	71.88	90.35	98.35	51.77	69.60	84.50	36.29	68.03	94.50

Bold values indicate a strong correlation.

During the winter, the first factor accounted for 36.3% and 26.35% of the total variance for particulate- and gaseous-phase PAHs, respectively (Table 2). The higher molecular weight PAHs had reduced the loading concentration for gaseous-phase and particulate-phase PAHs. This factor was also dominated by Nap and Ace for gaseous-phase PAHs and Anth, Flt, and Chr for particulate-phase PAHs, which indicated a natural gas combustion source (71). The second factor accounted for 31.73% of the total variance for particulate-phase PAHs, and Phen and Pyr were dominant, which may have been emitted from diesel sources. The results obtained from PC-2 demonstrated that the low molecular weight (Phen) indicated a petrochemical source (77, 78). For the third factor, high molecular weight PAHs [B(b)F, B(a)P, and DahA] were dominant components, which accounted for 26.5% of the total variance for particulate-phase PAHs. Many researchers suggested that these species originated from vehicular sources, especially from diesel emissions (20, 79). Hence, the results of PCA revealed that the major source of PAHs was found to be vehicular emissions (diesel and gasoline) as well as wood burning (biomass burning).

**Table 2:** Result of factor analysis with varimax rotation for gaseous phase PAHs at different seasons.

**PAH**	**Summer**			**Monsoon**			**Winter**		
	**1**	**2**	**3**	**1**	**2**	**3**	**1**	**2**	**3**
Nap	0.636	−0.222	0.667	−0.264	**0.754**	0.380	0.778	0.198	0.424
Acy	0.659	−0.273	0.488	−0.340	**0.832**	−0.337	0.531	0.681	−0.166
Ace	0.441	−0.133	0.348	−0.178	**0.686**	−0.133	0.748	0.188	0.246
Flu	0.720	−0.499	−0.187	0.081	0.117	0.960	0.328	0.171	−0.591
Phen	0.585	0.110	−0.695	**0.875**	0.041	−0.099	0.194	0.583	−0.399
Anth	0.552	−0.148	−0.619	**0.910**	0.251	−0.081	−0.507	0.514	0.303
Flt	−0.179	0.359	0.100	0.772	0.181	0.150	−0.371	0.674	−0.046
Pyr	0.593	0.553	−0.175	0.677	0.281	−0.106	−0.374	0.393	0.670
BaA	0.280	**0.789**	0.212	–	–	–	−0.124	0.175	−0.013
Chr	0.332	**0.815**	0.139	**–**	–	–	−0.700	0.207	−0.332
Initial Eigenvalues	2.77	2.14	1.81	2.87	1.92	1.24	2.63	1.85	1.43
% of variance	27.69	21.45	18.10	35.89	24.01	15.58	26.33	18.53	14.30
Cumulative %	34.17	63.08	79.40	51.77	69.60	84.50	36.29	68.03	94.50

Bold values indicate a strong correlation.

## Correlation analysis for PAHs

Pearson's correlation was used to provide the correlation coefficients needed for data analysis, with a significant level of *p* < 0.05. The correlation of the total particulate- and gaseous-phase PAHs is presented in Tables 3, 4. A strong significant positive correlation was observed between B(ghi)P and DahA (0.92), B(a)P and IcdP (0.85), and B(a)P and DahA (0.821). The highest molecular weight was linked with particulate-phase PAHs and was released mainly from vehicular emissions. In addition to this, the correlation between Nap and Flu, Flu and Flt, and B(a)P and IcdP showed a moderate to high positive correlation ranging from 0.68 to 0.75 for particulate-phase PAHs. For the gaseous phase, a strong and positive correlation coefficient of 0.678 was observed between Acy and Nap, Nap and Ace. The lowest molecular weight emission was found in the gaseous phase as an indicator of petroleum sources (78). Furthermore, the correlation between Phe and Flu, Phe and Pyr, and Flu and Ant showed a low-to-moderate correlation ranging from 0.28 to 0.50 for gaseous-phase PAHs. A weak correlation of these gaseous-phase PAHs showed negligible sources of emissions.

**Table 3:** Correlation of PAH species in the particulate phase PAHs.

	**Nap**	**Acy**	**Ace**	**Flu**	**Phe**	**Ant**	**Flt**	**Pyr**	**B(a)A**	**Chry**	**B(b)F**	**B(k)F**	**B(a)P**	**IcdP**	**DahA**	**B(ghi)P**
Nap	1															
Acy	0.598^**^	1														
Ace	0.041	−0.175	1													
Flu	0.688^**^	0–0.734^*^	0.210	1												
Phe	0.079	0.088	0.010	0.414^*^	1											
Ant	−0.007	0.532^**^	0.328	−0.513^**^	−0.415^**^	1										
Flt	0.348^*^	0.305	0.387	0.638^**^	0.211	0.174	1									
Pyr	−0.102	0.050	0.784	−0.039	0.331^*^	−0.264	−0.125	1								
B(a)A	−0.034	−0.322	0.066	−0.056	−0.386^**^	−0.240	−0.499^**^	0.295^*^	1							
Chry	−0.191	0.510^**^	0.838	−0.385^*^	0.196	0.038	−0.013	0.275	−0.079	1						
B(b)F	0.029	0.397^*^	0.989^*^	−0.587^**^	−0.422^**^	0.134	−0.484^**^	0.107	0.689^**^	0.262	1					
B(k)F	−0.120	0.351	−0.246	−0.512^**^	0–0.718^**^	0.305^*^	−0.155	−0.248	0.450^**^	0.215	0.637^**^	1				
B(a)P	−0.233	0.056	−0.466	−0.463^*^	−0.552^**^	0.078	−0.304^*^	0.310^*^	0.620^**^	0.205	0.614^**^	0.731^**^	1			
IcdP	−0.241	0.336	−0.751	−0.515^**^	−0.331^*^	0.026	−0.224	0.259	0.425^**^	0.507^**^	0.523^**^	0.756^**^	0.851^**^	1		
DahA	−0.056	0.351	−0.908	−0.506^**^	−0.494^**^	0.423^**^	−0.054	0.300^*^	0.289^*^	0.156	0.413^**^	0.567^**^	0.821^**^	0.638^**^	1	
B(ghi)P	−0.097	0.356	0.598	−0.476^*^	−0.473^**^	0.495^**^	−0.035	0.294^*^	0.326^*^	0.153	0.456^**^	0.558^**^	0.753^**^	0.591^**^	0.922^**^	1

** Correlation is significant at the 0.01 level (two-tailed).

* Correlation is significant at the 0.05 level (two-tailed).

Bold values indicate a strong correlation.

**Table 4:** Correlation of PAH species in the gaseous phase PAHs.

	**Nap**	**Acy**	**Ace**	**Flu**	**Phen**	**Anth**	**Flt**	**Pyr**	**BaA**	**Chr**
Nap	1									
Acy	0.619^**^	1								
Ace	0.591^**^	0.481^**^	1							
Flu	0.196	0.343^**^	0.257^*^	1						
Phen	−0.054	0.048	0.041	0.227	1					
Anth	−0.057	0.129	−0.132	0.258	0.462^**^	1				
Flt	−0.066	0.076	−0.162	0.078	0.124	0.293^*^	1			
Pyr	−0.115	−0.165	−0.080	−0.197	0.351^**^	0.301^*^	−0.015	1		
BaA	0.056	−0.011	0.017	−0.032	0.129	0.170	−0.100	0.011	1	
Chr	−0.407^**^	−0.186	−0.286	−0.080	0.195	0.007	0.398^*^	0.471^**^	0.060	1

** Correlation is significant at the 0.01 level (two-tailed).

* Correlation is significant at the 0.05 level (two-tailed).

Bold values indicate a strong correlation.

## Toxicity of PAHs

Emission sources of air pollutants, especially PAHs, play a significant role in understanding and determining their potential in environmental and human health assessment. This study estimated the potential toxicity of human exposure to all selected sites in Delhi in terms of total TEFs. Total TEQ values at all sites were calculated to be 38.39 and 0.55 ng/m^3^ for particulate- and gaseous-phase PAHs, respectively. The largest contributor to the total risk of particulate-phase PAHs was estimated to be D(ahA) (43.66–45.42%), followed by B(a)P (34.62–44.31%) at all sites, which was similar to the study conducted in Pakistan for D(ah)A (42.52–80.91%) followed by B(a)P (4.42–35.51%) in all cities (80). The maximum TEQ value for particulate-phase PAHS was attributed by D(ahA) at JNU (7.46), Mukherjee Nagar (20.94), Anand Vihar (18.99), and CP (18.28). Similar TEQ values at Anand Vihar and CP for particulate-phase PAHs were highly predominant in traffic areas, which indicated a similar source of emission driven by diesel- and gasoline-powered vehicles, whereas Mukherjee Nagar was considered to have a high population density and local emission sources such as wood and charcoal burning for cooking contributed to higher PAH concentrations. The current study focused on evaluating the health risk assessment in terms of LADD and cancer risk due to exposure to both particulate-phase and gaseous-phase PAHs. Average LADD values for children and adults were calculated as 3.17 × 10^−6^, 1.65 × 10^−6^ and 1.18 × 10^−6^, 6.12 × 10^−6^ for particulate-phase and gaseous-phase PAHs, respectively. In the gaseous phase, LADD values were reported to be relatively higher for adults than for children. The reason could be that gasoline was a significant source of gaseous-phase PAHs and adults are usually exposed to these for longer periods. In the particulate phase, the LADD value was higher for children, as a major source of particulate-phase PAHs was biomass burning, including wood burning in outdoor and indoor premises, to which children were more exposed. JNU observed minimum LADD values compared to other sites due to less movement of transport inside the campus. Cancer risk for children and adults at all sites was estimated for gaseous-phase and particulate-phase PAHs. The average value for cancer risk for children and adults were found to be 4.53 × 10^−6^, 2.36 × 10^−5^ and 1.22 × 10^−5^, 6.35 × 10^−5^ for particulate-phase and gaseous-phase PAHs, respectively, which indicated that the values were found to be much higher than the prescribed standard (1.0 × 10^−6^). Similar results were reported for children and adults, with 3.5 × 10^−5^ and 1.17 × 10^−5^ for the hot season and 3.30 × 10^−5^ and 1.10 × 10^−5^, respectively, for the hot and cold seasons (7).

## Conclusion

This study analyzed seasonal variations, source identification, and toxicity of PAHs in urban sites. The concentration of TPAHs (particulate and gaseous phases) in all monitoring sites was higher in the winter season (165.14 ± 50.44 and 65.73 ± 16.84 ng/m^3^) than in the summer season (134.08 ± 35.0 and 43.43 ± 9.59 ng/m^3^), whereas in the monsoon season, the concentration was lower (68.15 ± 18.25 and 37.63 ± 13.62 ng/m^3^). The main source of PAH emission was manmade sources, including the burning of wood and stubble burning during the winter from the neighboring states like Punjab and Haryana. Some emission sources of PAHs in Delhi certainly had large seasonal variations, including residential biofuel burning and open burning of biomass. To identify the source apportionment of PAHs through statistical tools, this study used PCA analysis and revealed that natural gas combustion was significantly attributed to the particulate-phase PAHs during the winter season, followed by diesel-driven vehicles in the ambient atmosphere of Delhi. During the summer season, vehicular emission was a major contributor of particulate-phase PAHs, followed by gasoline. In the case of the gaseous phase, PAH dominant species B(a)P and Chry may have been emitted from gasoline emission. This study can contribute to a better understanding of the monitoring of both particulate- and gaseous-phase PAHs in the ambient atmosphere of the urban area.

The current study focused on evaluating the health risks in terms of LADD and cancer risk due to exposure to both particulate- and gaseous-phase PAHs. Average LADD values for children and adults were calculated to be 3.17 × 10^−6^, 1.65 × 10^−6^ and 1.18 × 10^−6^, and 6.12 × 10^−6^ for particulate- and gaseous-phase PAHs, respectively. The average value of cancer risk for particulate- and gaseous-phase PAHs were found to be 4.53 × 10^−6^ and 2.36 × 10^−5^ for children but 1.22 × 10^−5^ and 6.35 × 10^−5^ for men at all monitoring sites, respectively, which indicated much higher values than the prescribed standard (1.0 × 10^−6^) by CPCB. The carcinogenic health risk for this study was reported to be relatively higher than the prescribed standard values (1.0 × 10^−6^). This study confirmed that the PAH levels in the ambient atmosphere of Delhi could not be neglected, and this study would be enlightening among the scientists, researchers, and government to address the issues along with policy formulation. Furthermore, this study can enhance policymakers with appropriate scientific solutions, such as imposing a ban on the burning of steeples, examining the quality of petroleum (petrol and diesel), and setting antipollution measures, whereas the issue of health risk assessment and recognition of factors affecting pollution is crucial and essential. Further, more comprehensive studies are required in this area. Thus, the results of this study emphasize the need for continuous monitoring of particulate- and gaseous-phase PAHs in the ambient air of Delhi, whereas the chances of exposure to the population are high for PAHs, which cause health risks such as cancer.

## Data availability statement

The original contributions presented in the study are included in the article/Supplementary material, further inquiries can be directed to the corresponding author.

## Author contributions

BS: conceptualization, analysis of the results and discussion, and visualization of graphs and tables. TZ: procurement of data. SA: frame the introduction section. AA-A: data curation and methodology. SR: conceptualization, results and discussion, and writing—review and editing. All authors contributed to the article and approved the submitted version.
