# Supplementary material for: Statistical analysis, source apportionment, and toxicity of particulate- and gaseous-phase PAHs in the urban atmosphere
Source: Front Public Health. 2023 Jan 10;10:1070663. doi: 10.3389/fpubh.2022.1070663 (PMC9871548; doi:10.3389/fpubh.2022.1070663)
Supplement: Supplementary file 1 [file Data_Sheet_1.docx]

**Supplementary file**

**Seasonal variation, source apportionment, and toxicity of particulate and gaseous phase PAHs in the urban atmosphere**

Bhupendra Pratap Singh^1,2^, Torki A. Zunhaibai^3,4^, Saif A. Alharthy ^3,4^, Ahmed I. Asmari^4,5^, Shakilur Rahman^6^

Stable 1: Geographical coordinates of selected sampling sites in Delhi

| S. No. | Location | Site Type | Geographical coordinates | |
| --- | --- | --- | --- | --- |
|  |  |  | Latitude | Longitude |
| 1 | JNU | Residential cum institution | 28.5458° N | 77.1703° E |
| 2 | Mukherjee Nager | Commercial cum traffic | 28.6353° N | 77.2249° E |
| 3 | Rohini | Residential cum traffic | 28.7373° N | 77.0909° E |
| 4 | Anand Vihar | Traffic | 28.6476° N | 77.3144° E |
| 5 | Connaught Place | Commercial | 28.6328° N | 77.2197° E |

Stable2. Meteorological parameters at different sites.

|  | **Summer** | | **Monsoon** | | **Winter** | |
| --- | --- | --- | --- | --- | --- | --- |
| **Sites** | **Temp °C** | **RH %** | **Temp °C** | **RH %** | **Temp °C** | **RH %** |
| JNU | 34.46±3.39 | 53.12±7.14 | 25.16±2.49 | 88.05±6.29 | 9.81±1.79 | 94.29±3.56 |
| Mukherjee Nagar | 34.26± 3.49 | 43.34±8.71 | 26.79±5.01 | 81.23±3.51 | 10.53±1.85 | 86.71± 4.29 |
| Rohini | 37.57± 4.81 | 49.25±7.91 | 28.94±1.69 | 78.14±7.59 | 10.98±1.04 | 81.23±3.96 |
| Anand Vihar | 39.02± 6.02 | 41.72± 8.47 | 28.90±3.65 | 76.21± 6.23 | 11.51± 3.65 | 76.12±4.26 |
| Connaught Place | 36.72± 5.41 | 46.17±7.32 | 28.92±3.51 | 77.98±5.34 | 12.68±2.39 | 74.98±3.92 |

**Method and Materials**

*Extraction and Chemical Analysis*

In the first stage, XAD-2 and the filter were kept at room temperature to get it warm. For extraction, XAD-2 resin tubes were broken from both ends, and cotton wool plugs were removed safely with the help of a twister. The resin of XAD-2 tubes was placed into 4 mL screw-top vials. The front and back sections of XAD-2 resin were placed in different vials and labeled as the front and back with a marker. The PTFE filter was first cut into small pieces, and they were also placed in separate 4mL screw-top vials. In each vial, 2 mL methyl chloride was added, and it was sacked for 2 minutes. These vials were allowed to settle for 30 minutes. Lab and field blanks were also extracted in the same way. From each vial containing XAD resin or filter, 1 mL extract was transferred to an autosampler vial for further analysis of GC/MS. The analysis was carried out on Bruker 450GC (Gas Chromatograph) equipped with capillary column DB-5 (30m x 0.25mm x 0.25µm film thickness). The analysis was performed according to the procedures listed by the National Institute for Occupational Safety Health (NIOSH) Method 5515 for the analysis of PAHs in air samples (NIOSH, 1994).

*Analytical Procedure*

*Chromatography analysis*

GC was used for the separation, identification, and quantification of the PAH compound. They were as follows: carrier gas was helium (1 mL/min). Injection mode was split with 100:1 (300 ^o^C, 1µL). The temperature program was started at 100^o^C, held for 4 min, then raised to 8 ^o^C per min up to 110 ^o^C and held for 5 min, a second heating rate of 5 ^o^C per min up to 290 ^o^C held for 35 min and finally a third heating rate of 1.5 ^o^C per min up to 320 ^o^C held for 5 min. The transfer line temperature was set at 280 ^o^C. The PAH in the chromatograms were identified by means of their retention times.

**Determination of analytical method characteristics**

Calibration is required for identification of PAHs by internal standardization. Response factors (Rf) is drawn by the calibration curve defined in equation 1.

***Calculation of the response factor:***

$$Rf=\frac{As X C[IS]}{A[IS] X Cs}$$

R*_f_* - response factor determined by the analysis of standard PAH and internal standard

A_s –_ area of the PAH peak in the calibration standard

A_[IS] –_ area of the internal standard peak for the calibration standard

Cs – PAH concentration for the calibration standard solution (ng/µl)

C_[IS]_ - internal standard concentration for the calibration standard solution (ng/µl)

Extracted sample was analyzed by the addition of internal standards to all calibration standards through GC. If the RF is constant over the working range (less than 20 percent RSD), assume the RF to be invariable and use the average RF for calculations.

It is suggested that check the calibration curve after every analysis of samples by measurement of one or more calibration standards. If the response for any parameter varies from the predicted response by more than ± 20 percent, it needs to recalibrate the standard curve.

***Sample analysis:***

Dilute the extracted residue and makeup to 0.5 ml or 1 ml. Inject 1 µl or 2 µl nto GC-FID for analysis and record the resulting peak size in area units I (or peak height when overlapping peaks cause errors in area integration). Identify the compounds using external calibration procedures from the sample chromatograms (Annexure)

***Calculations for each sample of the absolute amount of PAH that was extracted from the sample:***

$X\mathrm{PAH}=\frac{A\mathrm{PAH} X X[IS]}{A[IS] X Rf}$

X _PAH_ – absolute amount of PAH that extracted from the sample

A_PAH_ – area of PAH peak of the sample

A_[IS]_ - area of the internal standard peak of the sample

X_[IS]_ – absolute amount of internal standard added to the sample

Calculate the air volume from the periodic flow reading taken during sampling using the following equation

V = Average flow rate of sampling, m^3^/min x T

Where

V total sample volume at ambient conditions, in m^3^; and

T = elapsed sampling time, in min.s

The volume of air sampled (Vs) may optionally be converted to standard conditions of temperature and pressure (25°C and 101 kPa) using the following equation:

Vs= Vx (Pal 101) X [298/(273 + Ta)]

where

V = total sample volume under ambient conditions, in m3;

P a = ambient pressure, in kPa; and

Ta = ambient temperature, in °C.

The concentration, in nglm^3^, of each analyze in the air sampled is given by:

C = Cs X Ve / Vs

where

Ve = final volume of extract, in µl; and

Vs = volume of air sampled

Quality Assurance and Quality Control (QA/QC)

For quality assurance and quality control (QA/QC), three sets of XAD-2 tubes and three sets of filter blank spike and blank spike duplicate were spiked with 16 PAH spiking solution which is purchased from Sigma Aldrich (EPA 610 PAH mix, Catalog No. 48743, U.S.A.). Apart from this, the XAD-2 tube and filter paper were spiked with internal standard solution prior to extraction in order to check procedural performance and matrix effects (Semi volatile Internal Standard Mix, Catalog no. 48902. They were extracted and analyzed in the same way as the samples. All PAHs concentration efficiencies method detection limits were corrected for the 16 PAHs recoveries. No detection amounts of PAHs were found in any of the blank samples, with were extracted in the same way as the samples.

S fig 1: GC instrument for analysis of PAHs in air sample


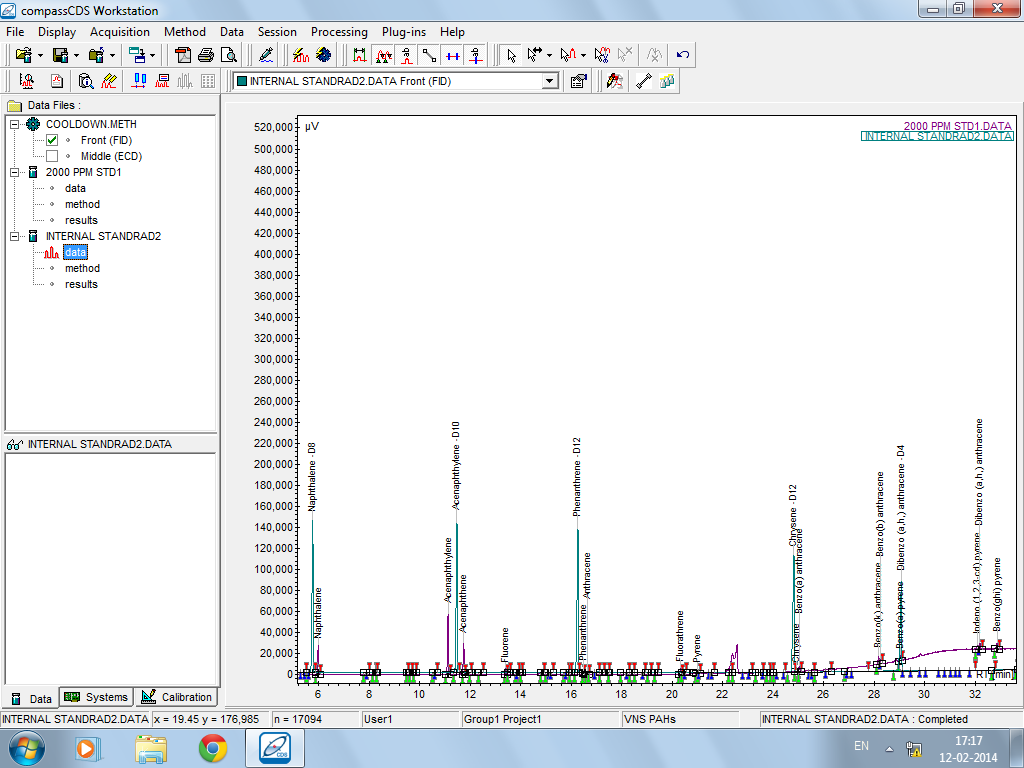


Sfig 2: Sample analysis with internal standard through GC for PAHs

***Health Risk Assessment***

According to USEPA, the TEFi values for PAHs such as Flu, Phe, Ant, Flua, Pyr, BaA, Chr, BbF, BkF, BeP, BaP, Per, IcdP, B(ghi)PiP, DahA, and Cor are 0.001, 0.001, 0.01, 0.001, 0.001, 0.1, 0.01, 0.1, 0.1, 0.01, 1,0.001, 0.1, 0.01, 1, and 0.01, respectively.

1) The incremental lifetime cancer risk (ILCR) was estimated as the exposure risk for chemicals suspected to have carcinogenic effects based on the USEPA standard models (USEPA, 2006; Chen and Liao, 2006; Peng et al., 2011). ILCR was calculated based on the corresponding lifetime average daily dose (LADD) of PAHs by considering two different age groups of children (age 6 years) and adults (age 70 years). LADD indicates the amount of PAH intake per Kg of body weight per day. Equations (2) and (3) were used for estimating LADD and ILCR, respectively.

LADD (mgkg^-1^day^-1^) = (Cs x IR x CF x EF x ED) / (BW x AT) Cancer risk (ILCR)

Cancer Risk = LADD x CSF (Slop Factor)

Where CS is the sum of converted concentrations of PAHs based on toxic
equivalents of BaP (ng m^-3^) using the Toxic Equivalency Factor (TEF) value. IR is the air inhalation rate (m^3^ day^-1^) (Peng, Zeng, & Chen, 2003), CF is the unit conversion factor (1 x 10^-6^ mg kg^-1^), EF is the exposure frequency (day year-1), ED is the exposure duration (day years^-1^) (Bartos, Cupr, Klánová, & Holoubek, 2009; Wang et al., 2011). ED is the exposure duration of 6 years for children and 52 years for an adult. BW represents the body weight (kg) (Peng et al., 2003). AT represents the averaging time for carcinogens (days) (US EPA, 2002), and CSF represents the inhalation cancer slope factor (3.85 mg kg-1 day-1). PEF represents the particle emissions factor (m^3^kg^-1^). CSFingestion, CSFDermal, and CSFInhalation of BaP were addressed as 7.3, 25, and 3.85 (mg kg^-1^ day^-1^)-^1^, respectively, determined by the cancer-causing ability of BaP (Peng et al., 2011). All the standard values were obtained from the US Environmental Protection Agency (2011).

**Results and discussion**

Stable 3. Principle component Analysis for annual the particulate phase PAHs annually.

| **Component Matrix^a^** | | | | | | |
| --- | --- | --- | --- | --- | --- | --- |
|  | Component | | | | | |
|  | 1 | 2 | 3 | 4 | 5 | 6 |
| Nap | -.355 | -.019 | -.218 | .469 | .442 | .227 |
| Acy | -.281 | .658 | -.051 | .409 | .306 | -.087 |
| Ace | .102 | .223 | -.568 | .115 | .342 | .408 |
| Flu | -.362 | -.257 | -.306 | .447 | -.558 | .294 |
| Phe | -.641 | .483 | .394 | -.029 | .073 | .203 |
| Ant | .303 | -.621 | .198 | .065 | .571 | -.250 |
| Flt | -.368 | -.453 | .363 | .448 | .000 | .354 |
| Pyr | .182 | .689 | .471 | .365 | -.164 | -.158 |
| B(a)A | .606 | .472 | -.438 | .089 | -.177 | -.156 |
| Chry | .293 | .245 | .605 | -.357 | .190 | .464 |
| B(b)F | .751 | .309 | -.312 | -.094 | .344 | .072 |
| B(k)F | **.838** | -.282 | -.211 | -.110 | -.008 | .280 |
| B(a)P | **.934** | .077 | .029 | .127 | -.240 | .033 |
| IcdP | **.834** | .115 | .192 | -.065 | -.171 | .370 |
| DahA | **.813** | -.171 | .253 | .397 | -.005 | -.082 |
| BghiP | **.806** | -.141 | .253 | .394 | .101 | -.148 |
| Initial Eigenvalues | 5.61 | 2.37 | 1.870 | 1.420 | 1.350 | 1.05 |
| % of Variance | 35.09 | 14.83 | 11.75 | 8.93 | 8.48 | 6.60 |

Stable 4. Principle component Analysis for annual the gaseous phase PAHs

| **Component Matrix^a^** | | | |
| --- | --- | --- | --- |
|  | Component | | |
|  | 1 | 2 | 3 |
| Nap | **.739** | -.436 | .160 |
| Acy | **.797** | -.324 | -.063 |
| Ace | **.674** | -.466 | .324 |
| Flu | **.674** | .017 | -.312 |
| Phen | .460 | .595 | .305 |
| Anth | .492 | **.663** | -.193 |
| Flt | .416 | .391 | -.568 |
| Pyr | .192 | **.628** | .596 |
| Initial Eigenvalues | 2.750 | 1.850 | 1.040 |
| % of Variance | 34.44 | 23.17 | 12.990 |
